# Generalized problematic internet use, emotional regulation and self-esteem in adults

**DOI:** 10.1192/j.eurpsy.2022.2110

**Published:** 2022-09-01

**Authors:** B.R. Maia, P. Morgado

**Affiliations:** 1 Universidade Católica Portuguesa, Faculty Of Philosophy And Social Sciences, Centre For Philosophical And Humanistic Studies, Braga, Portugal; 2 Universidade Católica Portuguesa, Faculty Of Philosophy And Social Sciences, Braga, Portugal

**Keywords:** generalized problematic internet use, adults, emotional regulation, self-esteem

## Abstract

**Introduction:**

Many internationally studies, in the last two decades, found problematic internet use associated with a variety of psychosocial problems, but in Portugal this is a recent research question specially in adults.

**Objectives:**

To explore the relationship between problematic Internet use, emotional regulation and self-esteem.

**Methods:**

138 Portuguese subjects (77.5% females), with a mean age of 27.76 years old (*SD* = 8.98, range: 18-58) filled in the Portuguese versions of the Generalized Problematic Internet Use Scale-2, the Difficulties in Emotion Regulation Scale and the Rosenberg Self-Esteem Scale.

**Results:**

Negative consequences subscale of generalized problematic internet use was positively correlated with all the emotional regulation difficulties subscales and negatively with Self-Esteem, and positively with daily hours of internet usage. A similar result was found for Self-Deficient Regulation subscale, except for Clarity subscale. Mood Regulation was correlated with Strategies, Goals and Self-Esteem. Males showed higher levels of Negative Consequences. Age and age onset of Internet use were negatively correlated with Mood Regulation, Self-Deficient Regulation and Negative Consequences. A statistically significant difference in Mood Regulation, Self-Deficient Regulation and Negative Consequences in marital status levels, and in professional situation, with higher median scores in divorced and single without a relationship and in student subjects; no significant differences were found in educational level.

**Conclusions:**

Generalized problematic Internet use, especially their Negative Consequences, is associated with higher emotional dysregulation, low self-esteem, lower age and lower age of Internet onset, being divorced or single without a relationship and being student, and it is more prevalent in males.

**Disclosure:**

No significant relationships.

